# Chemical Vapor Transport Synthesis of Fibrous Red Phosphorus Crystal as Anodes for Lithium-Ion Batteries

**DOI:** 10.3390/nano13061060

**Published:** 2023-03-15

**Authors:** Lei Liu, Xing Gao, Xuemei Cui, Bofeng Wang, Fangzheng Hu, Tianheng Yuan, Jianhua Li, Lei Zu, Huiqin Lian, Xiuguo Cui

**Affiliations:** 1 College of New Materials and Chemical Engineering, Beijing Institute of Petrochemical Technology, Beijing 102617, China; 2 School of Materials Science and Engineering, Beijing University of Chemical Technology, Beijing 100029, China; 3 Department of Mechanical and Materials Engineering, College of Engineering and Applied Science, University of Cincinnati, 2600 Clifton Ave, Cincinnati, OH 45221, USA; 4 Kailuan (Group) Limited Liability Corporation, Tangshan 064012, China

**Keywords:** fibrous phosphorus, lithium-Ion battery, anode

## Abstract

Red phosphorus (RP) is considered to be the most promising anode material for lithium-Ion batteries (LIBs) due to its high theoretical specific capacity and suitable voltage platform. However, its poor electrical conductivity (10^−12^ S/m) and the large volume changes that accompany the cycling process severely limit its practical application. Herein, we have prepared fibrous red phosphorus (FP) that possesses better electrical conductivity (10^−4^ S/m) and a special structure by chemical vapor transport (CVT) to improve electrochemical performance as an anode material for LIBs. Compounding it with graphite (C) by a simple ball milling method, the composite material (FP-C) shows a high reversible specific capacity of 1621 mAh/g, excellent high-rate performance and long cycle life with a capacity of 742.4 mAh/g after 700 cycles at a high current density of 2 A/g, and coulombic efficiencies reaching almost 100% for each cycle.

## 1. Introduction

With the growing urgency of the global energy crisis and environmental pollution, the development and application of clean energy must be vigorously promoted. Great progress has been made in sustainable energy technologies based on wind or solar energy. Therefore, it is desirable to develop efficient, safe and inexpensive energy storage technologies. Rechargeable (secondary) batteries are typically used in small to medium scale energy storage. Among these, LIBs have been utilized as the predominant power source of portable electronic devices due to their relatively high energy density, long life, lack of memory effects and environmental friendliness [1,2,3].

Recently, phosphorus (P) has turned out to be the most promising anode material candidate for LIBs with a high theoretical specific capacity (2596 mAh/g, based on the alloying process of P-LiP-Li_2_P-Li_3_P) [4]. The element phosphorus exists in three main forms: white phosphorus (WP), RP and black phosphorus (BP). Figure S1 shows the structures of different allotropes of phosphorus. WP is very unstable and flammable due to the weak bonding energy of tetrahedral P_4_, which is very dangerous and makes WP unsuitable for use in LIBs [5]. BP is also widely recognized for its high electrical conductivity (−100 S/m, close to hard carbon) and its application in LIBs [6], but the difficulty of preparation and the high price of BP severely limit the commercialization of BP. Compared to WP and BP, RP is a more prospective commercial anode material that is not only cheaper but also environmentally friendly. However, there are two main challenges limiting the practical application of RP: (1) RP has an extremely low electronic conductivity of 10^−14^ S/m and is almost non-conductive. (2) RP suffers from a huge volume expansion of about 300% during cycling [5,7,8,9].

These problems can lead to material fracture and pulverization of the red phosphorus material during cycling, as well as rapid capacity decay. To address these problems, many researchers have demonstrated the need to build microstructures and reduce the size of the active material to achieve fast kinetics. Park et al. [10] prepared phosphorus–carbon composites by high-energy ball milling, which significantly improved the electrical conductivity of P and enabled its application in LIBs. Zhou et al. [11] successfully prepared hollow phosphorus nanospheres with porous shells and controlled diameters by a solvothermal method. The hollow nanosphere structures of these porous shells effectively adapt to volume changes and avoid the pulverization of active material, exhibiting excellent long-cycle performance in LIBs.

Wang et al. [12] first compounded porous carbon with RP by the evaporation–condensation method, resulting in a stable battery cycle of 55 turns. The evaporation–condensation method is considered to be an effective strategy to address the large volume expansion caused by conductivity and lithiation. However, there are two important issues in the evaporative condensation approach; firstly, the low P mass loading (~30 wt%) in the carbon-based framework severely limits the energy density. Secondly [13,14], the residual WP can lead to safety issues of flammability and high toxicity [15,16]. To address the low P mass loading and safety issues, Zhang et al. [17] systematically investigated the interactions between P_4_ and various functional groups in carbon materials (C_surf_, C_edge_, C−N−5, C−N−1, C−N_g_, C–S−5, C−S−6, C−S−6, C=O, C−O−C, C−OH and COOH) and provided a structural design strategy for the carbon framework to build up the edge sp^2^ carbon atoms. These edge carbon atoms, in turn, provide high adsorption energy for P_4_ molecules through the strong P-C bonds. The RP-PC anodes they prepared exhibit extremely high P mass loading (close to the theoretical limit for loading), strong and stable P-C bonds, and significantly improved electron and Li^+^ transfer, resulting in excellent high-rate, long-cycle stability.

Inspired by this previous work, we prepared FP materials by the CVT method in order to further improve the rate capability of P to Li storage. FP materials possess many electrochemical advantages: (1) the electrical conductivity of FP is 10^−4^ S/m (0.2 V), which is 8 orders of magnitude higher than that of commercial red phosphorus (RP) (10^−12^ S/m), and FP possesses better electrical conductivity compared to RP [18]. (2) FP is a 1-dimensional (1D) material, but it also possesses a 2-dimensional (2D) layered structure, and this 2D structure can effectively reduce the Li^+^ diffusion length and also enrich its electrochemical active center [19]. (3) FP can be reduced in size to the nanoscale by liquid phase exfoliation, and its small-enough size can effectively reduce the volume change of the material during cycling without structural damage [20]. The fibrous red phosphorus–graphite (FP-C) composite, prepared by FP with graphite by ball milling exhibited excellent cycling stability and high-rate performance, maintaining a high reversible specific capacity of 742.4 mAh/g after 700 cycles at high current densities.

## 2. Materials and Methods

### 2.1. Preparation of FP

(1) Synthesis of the FP: 6 g of commercial amorphous red phosphorus and 0.6 g of iodine were loaded into a quartz tube while maintaining a vacuum inside the tube. The tube was placed in a muffle furnace. The muffle furnace increased the temperature to 500 °C at a rate of 2.5 °C per minute and maintained it for 24 h, followed by natural cooling to room temperature. The quartz tube was broken, and the lump of FP was removed and ground into a powder, which was subsequently cleaned and dried using ethanol and acetone.

(2) Control experiment: 6 g of commercial amorphous red phosphorus was loaded into a quartz tube while maintaining a vacuum inside the tube. The subsequent heat and treatment process is as described above.

(3) Preparation of crystalline red phosphorus nanoribbons (FP NR): The cleaned FP powder was added to 250 mL of NMP and sonicated (600 W) for 12 h to disperse the FP in the NMP.

### 2.2. Preparation of FP-C and RP-C

Synthesis of the phosphorus–graphite composites: The phosphorus–carbon composites were prepared by a simple ball milling process. FP or RP was mixed with graphite powder (mass ratio 7:3), 1 g of the mixture was removed and added to zirconia (25 mL) and zirconia ball milling beads were placed (mass ratio 90:1). The above operations were carried out in a glove box. The jar was placed in a planetary wheel ball mill with a speed of 500 r/min and ball milling was carried out without interruption for 12 h.

### 2.3. Measurement of Material Characteristics

Analysis of the crystal phase of the material was carried out by X-ray diffraction (XRD) using a Panalytical Empyrean diffractometer. Raman spectra of the analyzed materials were collected using a Renishwa Raman instrument using a 532 nm laser wavelength. Analysis of the chemical composition of the material was determined using X-ray photoelectron spectroscopy (XPS), carried out on a Thermo ESCALAB 250XI using a monochromatic Al-Ka source (1486.6 eV). Observation of the morphology and structure of the prepared materials was carried out using a scanning electron microscope (SEM) and transmission electron microscopy (TEM). SEM images were collected using a ZEISS Gemini 300 device and TEM images were gathered using a JEOL JEM-F200 device operating at 200 kV.

### 2.4. Electrochemical Characterization of Materials

A CR2032 button cell, consisting of a phosphorus electrode, diaphragm and lithium metal, was assembled in an argon glove box with a glove box water oxygen content of less than 0.01 PPm. Preparation of the phosphorus based electrode: A homogeneous slurry was made by mixing phosphorus–carbon composite, acetylene black and polyvinylidene fluoride (PVDF) binder in a mass ratio of 7:2:1 into a N-Methyl pyrrolidinone (NMP) solution. The slurry was coated onto copper foil and baked in a vacuum drying oven at 120 °C for 12 h. The mass loading of electrodes was around 1.9 mg. A common electrolyte was used, 1 M/L LiPF_6_ at EC/DEC/EMC = 1:1:1, v/v, 10% FEC. Celgard 2400 film was used as the separator. Constant current charge/discharge tests were carried out using a LANDHE cell test system with a voltage window ranging from 0.01 to 2.5 V (for Li electrodes). Cyclic voltammetric curve testing was carried out using a CH1660B electrochemical workstation with an electrochemical window ranging from 0.01 to 3.0 V. Electrochemical impedance spectra were tested on a CH1660B electrochemical workstation in the frequency range from 10^5^ to 0.01 Hz. All of these electrochemical performances were tested under ambient temperature.

## 3. Results and Discussion

### 3.1. Characterization of FP

**Figure 1:**
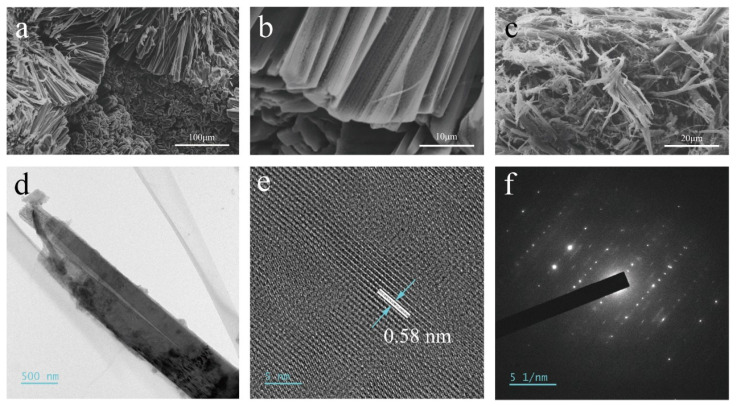
(**a**,**b**) SEM images of FP bulk; (**c**) SEM image of FP powder; (**d**) TEM image of FP NR; (**e**) HR-TEM image of the FP NR; (**f**) SAED pattern of a single of FP NR.

We prepared bulk FP by the low-temperature CVT method. As can be seen in Figure S2b, the addition of iodine (I_2_) causes the RP to nucleate and crystallize in the low-temperature region of the quartz tube to form FP blocks that attach to the inner wall of the quartz tube. This suggests that I_2_ plays a transport and catalytic role throughout CVT. RP as a solid source and I_2_ as a sublimed mineralizer produce gaseous intermediate iodophosphorus compounds (PI_x_) in the sublimation zone when the temperature reaches the sublimation temperature, which are transported towards the deposition zone by the PI_x_ partial pressure gradient [21]. The gaseous intermediate is converted to deposit products (FP) and releases mineralizer molecules. The released mineralizer molecules increase the partial pressure of the mineralizer in the deposition zone, thus they can transport back to the sublimation zone for further reactions until all the RP is converted to FP [22]. The bulks of FP (Figure S3) were ground and cleaned by ultrasonication with alcohol to remove I_2_ and dried by filtration to obtain FP powder. As shown in Figure S4, from optical microscope images of RP and FP powders, it is evident that RP is a deep red color, while FP appears orange-red. It can be seen that RP is a particle of approximately 40 µm in size, in contrast to FP which is a strip of fibers. There is a clear morphological difference between RP and FP, and RP can induce a transition from granular RP to one-dimensional fibrous FP after catalysis by I_2_. The FP was observed by SEM, as can be seen in Figure 1a, and the bulk FP after growth by CVT consisted of 50 µm long micron rods arranged neatly and showing a dispersive shape. A partial magnification of one end of one of the micrometer rods shows that the FP has a lamellar structure (Figure 1b). After ultrasonic exfoliation, the length and diameter of the FP powder were significantly shortened, both consisting of rods of a few tens of microns (Figure 1c). The morphology and structure of the FP nanoribbons were observed by TEM (Figure 1d), and it was found that the FP nanoribbons showed a lamellar shape. In the high-resolution TEM (HR-TEM) image (Figure 1e), the lattice stripe spacing of 0.58 nm corresponded to the (001) plane in the vertical view. In addition, the surface of the FP nanoribbons is locally oxidized, which is consistent with the P-O bonding results appearing by XPS. The selected area electron diffraction (SAED) pattern of a single nanoribbon in Figure 1f shows that single-crystal FP nanoribbons were formed along the [1$\bar{4}$1] axis.

**Figure 2:**
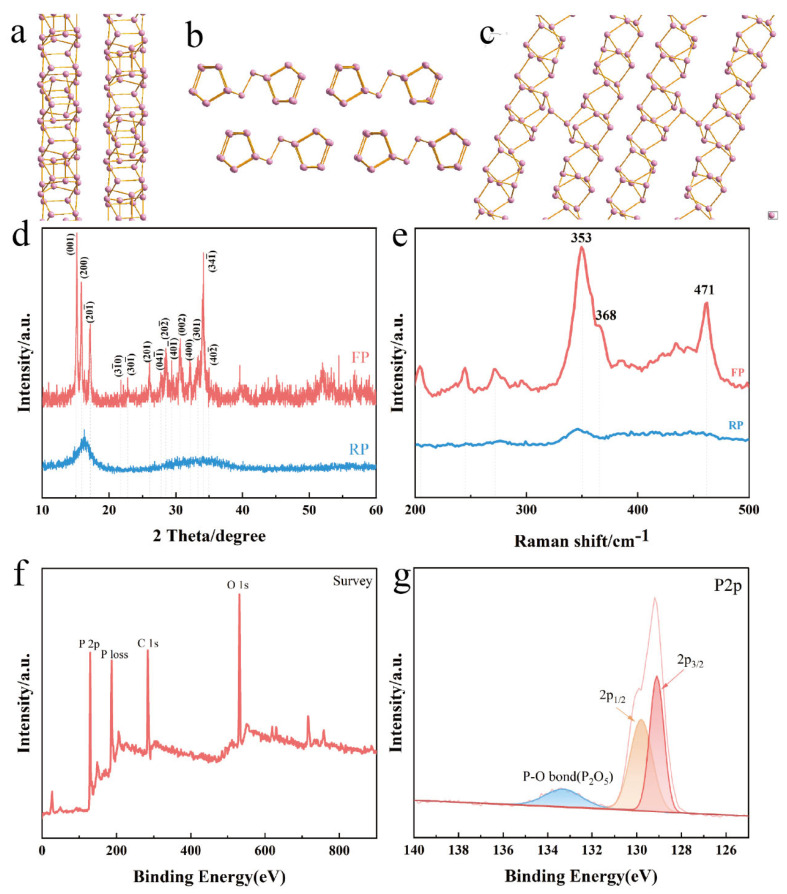
Schematic structural view of FP along the a (**a**), b (**b**) and c (**c**); (**d**) XRD curves and (**e**) Raman spectra of RP and FP; (**f**) XPS survey spectrum of FP; (**g**) P2p spectrum of FP.

FP was further compared with RP by XRD, as shown in Figure 2a. The XRD pattern of RP is a broad-peaked amorphous phase in the range of 12–18° and 26–36°, corresponding to the characteristic peaks of commercial amorphous RP. The XRD peak of FP corresponds highly to the XRD diffraction peak of triclinic P. Also based on the study of Du et al. [23] for FP, it shows that the sample we prepared was 1D FP. In their study of FP, Liu et al. [19] found that FP also has a 2D band structure with a nanoribbon thickness of approximately 7.8 nm, which indicates that FP has not only a 1D structure but also a 2D band structure. As can be seen from the structural diagram of FP (Figure 2a–c), FP consists of P-cage chains of P_8_ and P_9_ interconnected to form tubular structures, and the layers of these tubular structures are parallel to each other, with two tubular structures connected by two dumbbell-shaped p-atom structures. It should be noted that if the layers of the tubular structure are perpendicular to each other they are Hittorf’s phosphorus (HP) or violet phosphorus (Figure S1), which also results in a high degree of similarity between the XRD and Raman spectrum of FP and HP [18]. Moreover, based on Winchester et al.’s summary [24], the preparation of FP did not match with type II RP and matched best with type IV RP. Figure 2b contrasts the Raman spectra of FP and RP in the frequency range of 200–500 cm^−1^. It can be seen that RP has no obvious characteristic peaks, and only broad peaks appear in the range of 345–365 cm^−1^, which mainly comes from the long-range disordered structure of polymeric phosphides, while the Raman spectra of FP show complex vibrational patterns, mainly due to the low symmetry of trigonal crystals and the diversity of atomic coordination. The peak at 368 cm^−1^ is the stretching vibration of the P_8_ phosphorus cage and the peak at 353 cm^−1^ is the stretching vibration peak of the P_9_ phosphorus cage [25]. XPS was used to perform compositional analysis of the FP powders. As shown in Figure 2c, the presence of element I_2_ was not detected in the XPS survey spectrum, suggesting that the addition of I_2_ only facilitated the transition from RP to FP and did not adulterate the FP. In addition, the 2p^1/2^ and 2p^3/2^ peaks at 130.0 eV and 131.4 eV, respectively, correspond to the P-P bond [18]. The peak at 134.7 eV corresponds to the P-O bond (Figure 2d), which indicates that FP undergoes oxidation in air, which is identical to the oxidative nature of BP [26,27].

### 3.2. Electrochemical Properties of FP-C and RP-C

**Figure 3:**
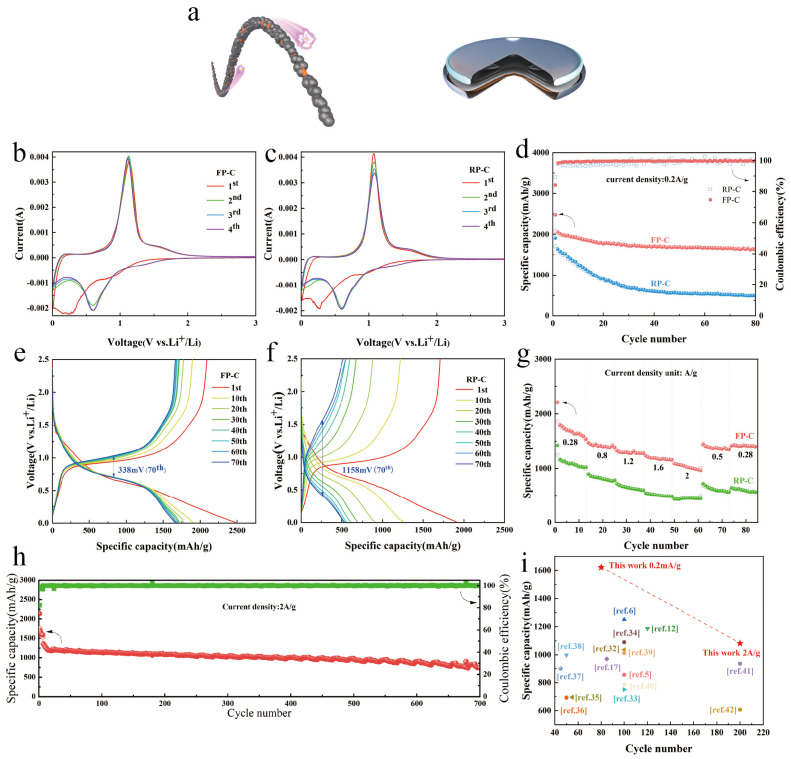
Electrochemical performance of RP-C and FP-C electrodes for LIBs. (**a**) Schematic illustration of the FP-C and button cell. (**b**,**c**) CV of the FP-C and RP-C anodes with a scan rate of 0.2 mV/s between 0.01 and 3.0 V vs. Li^+^/Li. Cycling stability of FP-C and RP-C anodes for 80 cycles at 0.2 A/g (**d**) and the corresponding voltage profiles of FP-C (**e**) and RP-C (**f**). (**g**) Rate performance of FP-C and RP-C anodes at the varied rate from 0.28 A/g to 2 A/g. (**h**) Capacity and coulombic efficiency during cycling of the FP-C anode at 2 A/g after 5 initial cycles at a low current density of 0.2 A/g. Overall capacity and cycling stability compared with other reported RP- and BP-based anode materials (**i**). The data and current density of each material are summarized in Table S1 (Supporting Information).

FP-C and RP-C composites were prepared by ball milling FP and RP with graphite and characterized by XRD and Raman (Figure S5), and the electrochemical properties of the composites were studied. The lithiation and de-lithiation processes of FP-C and RP-C composites under half-cells were investigated by cyclic voltammetry, tested at a scan rate of 0.2 mV/s with a voltage window of 0.01–3 V. As shown in Figure 3b,c, a broad peak appeared at about 1 V during the first cathodic scan of the CV curve of FP-C, which is attributed to the formation of the SEI film of the solid electrolyte [28], and the subsequent broad peak is the lithiation process of the P-based material. Two peaks of approximately 1.1 V and 1.6 V appeared in the anode, corresponding to a de-lithiation process of the P-based material [29,30]. In the subsequent cycles, the cathode peak shifted to 0.6 V and the peak area increased, followed by a high overlap of the CV curves, indicating the high reversibility and stability of the FP-C negative electrode. In contrast, the anodic peak of the RP-C negative electrode decayed severely and the peak area decreased, which suggests severe polarization and low reversible stability of the RP-C negative electrode. It is noteworthy that the redox peak potentials of FP and RP are almost identical, which illustrates that the electrochemical properties of FP and RP as anode materials in LIBs are highly similar, but the electrochemical properties owing to their different structures show great differences.

Figure 3d compares the cycling stability performance of FP and RP. FP-C shows excellent cycling stability and still holds a high specific capacity of 1621 mAh/g after 80 cycles, whereas the capacity of RP-C only remains at 494 mAh/g. The capacity of the RP-C electrode drops rapidly before 40 cycles, which is attributed to a rupture of the SEI film due to the huge volume expansion of RP during lithiation. The thicker SEI film creates such electrochemical segregation that the Li^+^ in the formed Li-P alloy cannot return to the Li metal to form dead Li^+^, while the larger volume expansion leads to pulverization of the active material, and this part of the P is no longer involved in the normal lithiation/delithiation process. The FP, however, may have a special 1D structure and nanoscale scale such that it does not continue to expand after a certain range of expansion [20], which results in the FP maintaining a very high reversible specific capacity after 80 cycles. It was observed that FP-C maintained a stable and high coulombic efficiency throughout the cycle, while the coulombic efficiency of RP-C fluctuated remarkably, which was attributed to the stable structure of FP-C (Figure 3a). The structural stability of the composite facilitates the stabilization of the SEI passivation layer during cycling (Figure S6), maintaining nearly 100% coulombic efficiency and excellent cyclability. Figure S6 shows that the FP remains a relatively intact structure after 20 cycles at a current density of 0.5 A/g. Figure 3c,f show the constant current charge/discharge curves for FP-C and RP-C at a current density of 0.2 mA/g. The hysteresis of the charge-discharge curve of RP-C increased significantly after cycling, rising to 1158 mV after 70 cycles, whereas the overpotential of FP-C was more stable and much lower than that of RP-C, at 338 mV after 70 cycles, a phenomenon that is highly consistent with the results of the CV curve.

The high-rate capability is also an important indicator to evaluate the performance of the battery. Figure 3g displays that FP-C exhibits a higher-rate capability than RP-C in the current density range of 0.28–2 A/g. Specifically, the average reversible capacities of FP-C at current densities of 0.28, 0.8, 1.2, 1.6, 2, 0.5 and 0.28 A/g were 1726, 1444, 1312, 1203, 1060, 1316 and 1638 mAh/g, respectively, while the average reversible capacities of RP-C were only 1116, 839, 661, 591, 449, 609 and 599 mAh/g, respectively. The contrast between the two is remarkable, as the reversible discharge capacity of RP-C is only 45% that of FP-C at a high current density of 2 A/g. After high multiplication cycles, when the current density returned to 0.28 mA/g, FP-C still had a specific capacity of 1401 mAh/g compared to 567 mAh/g for RP-C, indicating that FP-C exhibits excellent high-rate performance and fast response kinetics. It is noteworthy that the initial discharged capacity of FP-C is higher than that of RP-C in both the cycling test of low current density and the high-rate capability test. This may be due to the fact that the layered structure of FP allows Li^+^ to be embedded in the layers before the alloy reaction, similar to the insertion of BP into the layers before the alloy reaction [31], so that Li^+^ can alloy with more P atoms, while the 1D structure of FP allows Li^+^ to pass through faster [32]. Figure 3h shows that the reversible discharge capacity of FP-C remains at 742.4 mAh/g after 700 cycles at a high current density of 2 A/g, demonstrating the excellent high rate cycling stability performance of FP-C. Figure 3i and Table S1 show how this work compares to previous work, with the FP-C electrode exhibiting excellent electrochemical performance [5,6,12,17,32,33,34,35,36,37,38,39,40,41,42].

We prepared BP-C composites using the same method and investigated their cyclic stability and high-rate capability. As can be seen from Figure S7, the remaining reversible capacity of the BP-C negative electrode after 80 cycles at a current density of 0.2 A/g is 1555.7 mAh/g, a lower rate than that of the FP-C (1621 mAh/g). However, BP-C performed well in terms of high-rate capability with an average reversible capacity of 1250.13 mAh/g at a high current density of 2 A/g, compared to 1060 mAh/g for FP-C, suggesting that FP has better cycling stability compared to BP, and BP has better high-rate performance. The higher conductivity (100 S/m) of BP also contributes to the better high-rate performance of BP, which can be attributed to the special 1D structure of FP that allows FP-C to exhibit better cycling stability. Although the conductivity of FP is 8 orders of magnitude better than that of RP, the conductivity of FP is still lower than that of BP, indicating that FP is more suitable for energy storage applications than BP, while BP is more suitable for power battery applications.

**Figure 4:**
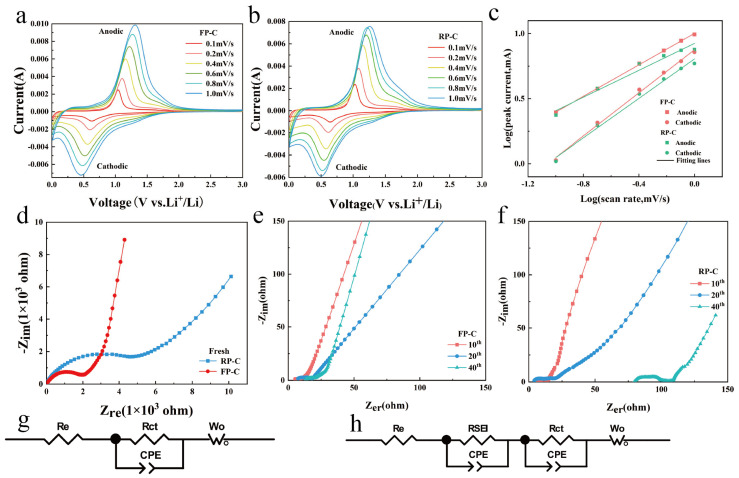
Electrochemical reaction kinetics of FP-C and RP-C electrodes for LIBs. CV curves of FP-C (**a**) and RP-C (**b**) at various scan rates from 0.1 to 1.0 mV/s for LIBs. (**c**) Log (peak current)-log (scan rate) curves for the observed cathodic and anodic peaks in (**a**,**b**). EIS Nyquist plots of fresh FP-C and RP-C electrodes (**d**). EIS Nyquist plots of FP-C (**e**) and RP-C (**f**) electrodes were tested after 10, 20, and 40 cycles at 0.2 A/g. Equivalent circuit (**g**) corresponding to (**d**) EIS spectra. (**h**) is the equivalent circuit for the EIS spectra of (**e**,**f**).

The kinetic factors were further investigated on the electrochemical performance by testing the CV of FP-C and RP-C at a series of scan rates of 0.1, 0.2, 0.4, 0.6, 0.8 and 1.0 mV/s, as shown in Figure 4a,b. The peak potentials of the CV curves for both FP-C and RP-C electrodes rise with increasing scan rate and cover the potential range of the lower scan rate, which also indicates the capacitive behavior of both FP-C and RP-C electrodes. According to equations 1 and 2 [36], there is a power law relationship between the peak current of the CV curve and the scan rate, and the electrochemical reactions can be divided into diffusion-Controlled interactions and capacitive processes.

i = aν^b^ (1)

log(i) = blog(ν) + log(a)(2)

where i is the peak current (mA), v is the scan rate (mV/s) and a and b are adjustable parameters.

As shown in Figure 4c, the b values for the FP-C anode and cathode peaks were calculated to be 0.604 and 0.827, respectively, indicating that both diffusion-Controlled alloying reactions and the capacitive processes dominate the electrochemical reactions of FP-C. Similarly, the b values of 0.516 and 0.757 for the anode and cathode of RP-C, respectively, suggest that the capacity of both FP-C and RP-C are diffusion-Controlled alloy reactions.

Electrochemical impedance spectroscopy (EIS) was also used to interpret the detailed electrochemical reaction kinetics. Figure 4d–f show the EIS spectra of FP-C and RP-C electrodes after testing at 0, 10, 20, and 40 cycles turns at a current density of 0.2 A/g. All Nyquist plots consist of a semicircle in the high-frequency region and a line in the low-frequency region, representing the charge transfer process and Li^+^ diffusion in the solid state, respectively. The equivalent circuits corresponding to the impedance data include the electrolyte and electrode ohmic resistance (R_e_), the SEI film resistance (R_SEI_), the charge-transfer resistance (R_ct_) and the parallel constant phase element (CPE), as well as the Warburg impedance (W_o_). The fresh RP-C electrode had an R_ct_ of ~4750 Ω, while the FP-C electrode had an R_ct_ of ~2035 Ω, showing the higher electronic conductivity of the FP-C. As shown in the graphs of turn 10 versus turn 20 of the cycle, the R_ct_ of both the RP-C and FP-C electrode decrease to a small stable value, indicating that a stable interface is formed between the electrolyte and the electrode (Figure 4e,f), while the R_ct_ of the RP-C is slightly larger than that of the FP-C. However, at the 40th cycle, the FP-C and RP-C show a more distinct difference, with the RP-C electrode having a higher internal cell resistance and being heavily polarized, corresponding to the cycle performance test in Figure 3d, which coincides with the time of maximum cell capacity decay. In the case of the FP-C electrode, the tendency for the battery capacity to decay decreases in 20 cycles and becomes more stable inside the battery at 40 cycles.

## 4. Conclusions

In conclusion, we have prepared FP by the low-temperature CVT method. FP significantly increases its electrochemical performance as a P anode electrode due to its special structure and high electrical conductivity. While FP was prepared with C by the ball milling method to produce FP-C composites, the composites exhibited much better electrochemical performance with excellent cycle stability and multiplicative properties than RP-C. The design strategy of FP-C offers great guidance implications for achieving mass-produced and high-performance P-based negative electrodes.

## Data Availability

The data presented in this study are available on request on request form the corresponding author.

## Supplementary Materials

The following supporting information can be downloaded at: https://www.mdpi.com/article/10.3390/nano13061060/s1. Table S1: Comparison of the electrochemical performance of our work with that of previous phosphorus-based LIBs. Figure S1. Schematic structures of different allotropes of phosphorus. Figure S2: Optical photographs of the quartz tube reactor after the reaction (a) without I2, (b) with I2. Figure S3: Optical photographs of FP bulk lumps. Figure S4: Optical photographs of filamentous red phosphorus. Figure S5: FP-C and RP-C composites’ (a) XRD diagram (b) Raman spectra. Figure S6. SEM images of the FP-C electrodes in LIBs after 20 cycles at a current density of 0.5 A/g. Figure S7: (a) Cycling stability of BP-C anodes for 80 cycles at 0.2 A/g. (b) Rate performance of FP-C and RP-C anodes at the varied rate from 0.28 A/g to 2 A/g.
