# Supplementary material for: Chemical Vapor Transport Synthesis of Fibrous Red Phosphorus Crystal as Anodes for Lithium-Ion Batteries
Source: Nanomaterials (Basel). 2023 Mar 15;13(6):1060. doi: 10.3390/nano13061060 (PMC10056364; doi:10.3390/nano13061060)
Supplement: Supplementary file 1 [file nanomaterials-13-01060-s001.zip › nanomaterials-2283232-supplementary.pdf]

Chemical Vapor Transport Synthesis of Fibrous Red Phosphorus Crystal as Anode for Lithium Ion Battery

Lei Liu[1,†], Xing Gao[2,†], Xuemei Cui[3], Bofeng Wang[1], Fangzheng Hu[1], Tianheng Yuan[1], Jianhua Li[4], Lei Zu[1,*], Huiqin Lian[1] and Xiuguo Cui[1,*]

[1] College of New Materials and Chemical Engineering, Beijing Institute of Petrochemical Technology, Beijing 102617, China
[2] School of Materials Science and Engineering, Beijing University of Chemical Technology, Beijing 10002, China
[3] Department of Mechanical and Materials Engineering, College of Engineering and Applied Science, University of Cincinnati.2600 Clifton Ave, Cincinnati, OH, 45221.
[4] Kailuan (Group) Limited Liability Corporation, Tangshan 064012, China
* Correspondence: zulei@bipt.edu.cn (Z.L.) ; cuixiuguo@bipt.edu.cn (C.X.)
† Lei Liu and Xing Gao authors contributed equally to this work.

Table S1. Comparison of the electrochemical performance of our work with that of previous phosphorus-based lithium-ion batteries.

Modified strategy	Current density	Cycle number	Residual capacity	Ref.
BP-G	0.2C	100	855mAh/g	5
PANI-(BP-C)	520mA/g	100	1250mAh/g	6
RP-porous carbon	260 mA/g	120	1187.7mAh/g	12
RP-sulfur/carbon	200 mA/g	200	935.3mAh/g	17
RP/CMK-3	0.25C	85	971mAh/g	32
BP/C/CNTs	150 mA/g	100	1036mAh/g	33
BP@NCNTs	100 mA/g	100	750mAh/g	34
RP/hierarchical micromesoporous carbon nanospheres	500 mA/g	100	1090mAh/g	35
RP/porous C	100 mA/g	55	696mAh/g	36
RP-rGO	100 mA/g	50	693mAh/g	37
RP/TiN/CNT	200 mA/g	45	900mAh/g	38
RP/CNT	0.5C	50	998.5mAh/g	39
P inside MWCNTs	100 mA/g	100	1012mAh/g	40
BP/RGO sandwiches film	100 mA/g	200	607mAh/g	41
Phosphorus@ZIF-8 Derived Porous Carbon	100 mA/g	100	786mAh/g	42
FP-C	200 mA/g	80	1621mAh/g	This work
FP-C	2A/g	200	1081.58mAh/g	This work

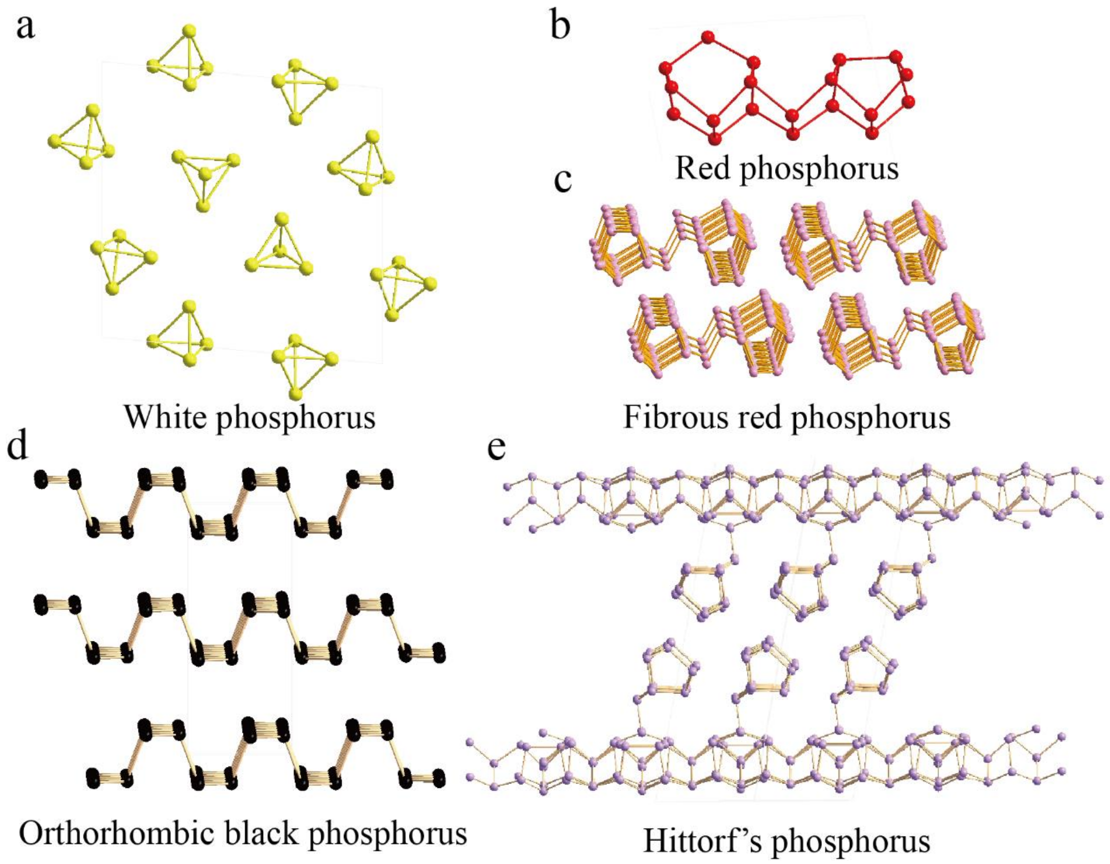

Figure S1. Schematic structural of different allotropes of phosphorus.

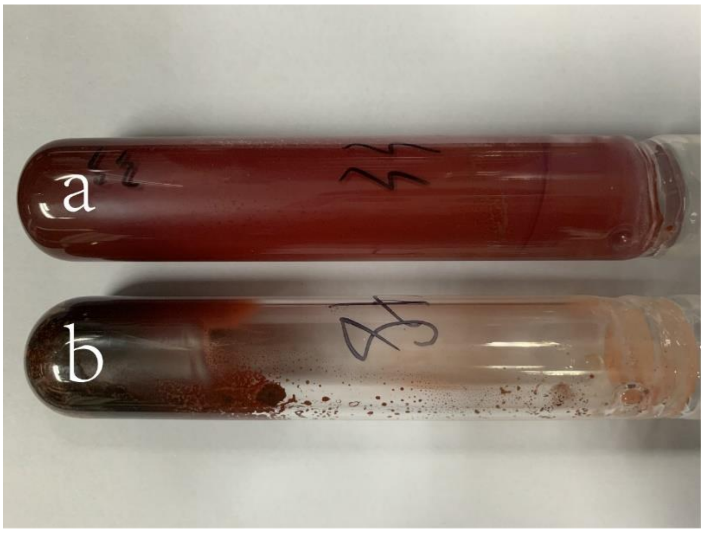

Figure S2. Optical photographs of the quartz tube reactor after the reaction. (a) without I_2, (b)with I_2.

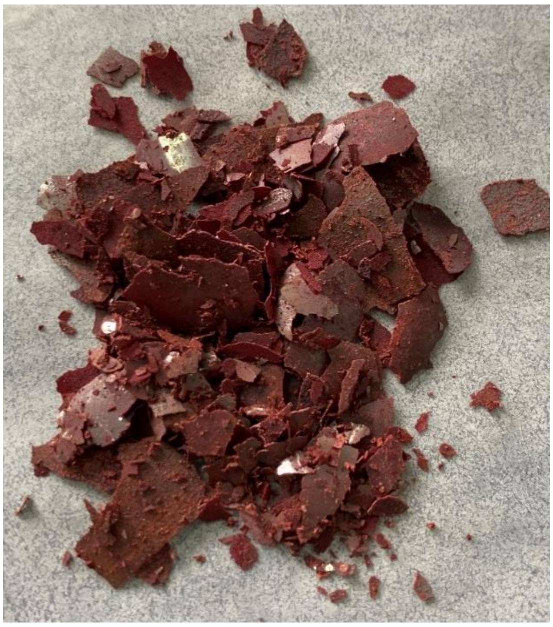

Figure S3. Optical photographs of FP bulk lumps.

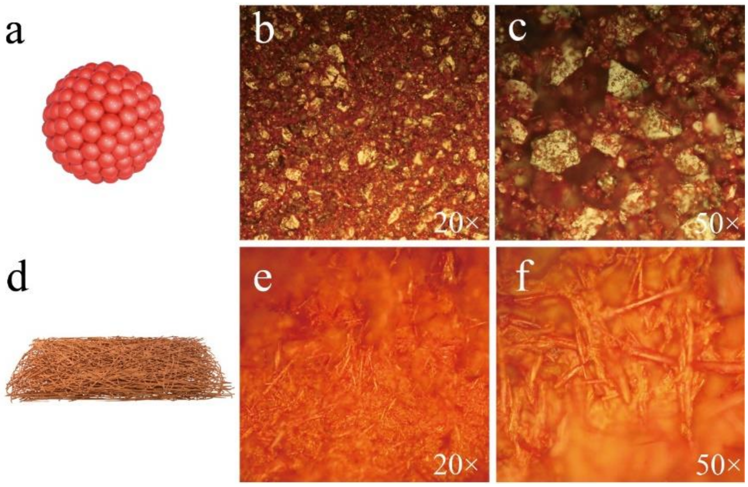

Figure S4. Optical photographs of Filamentous red phosphorus.

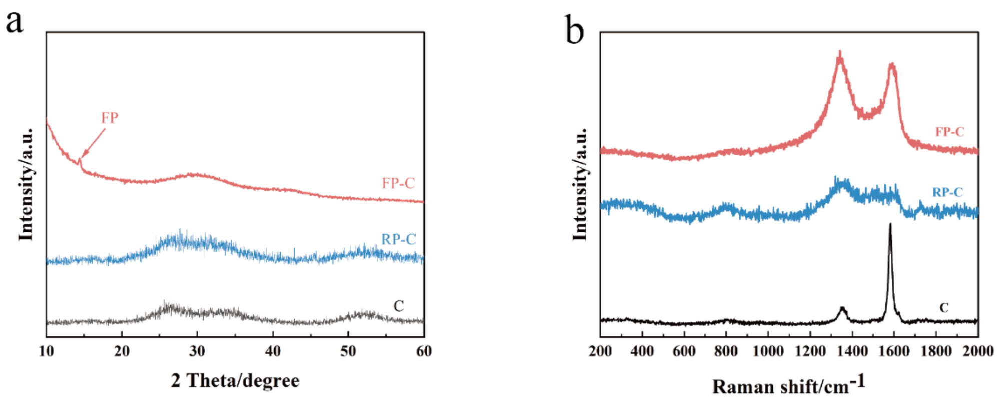

Figure S5. FP-C and RP-C composites (a) XRD diagram (b) Raman spectra.

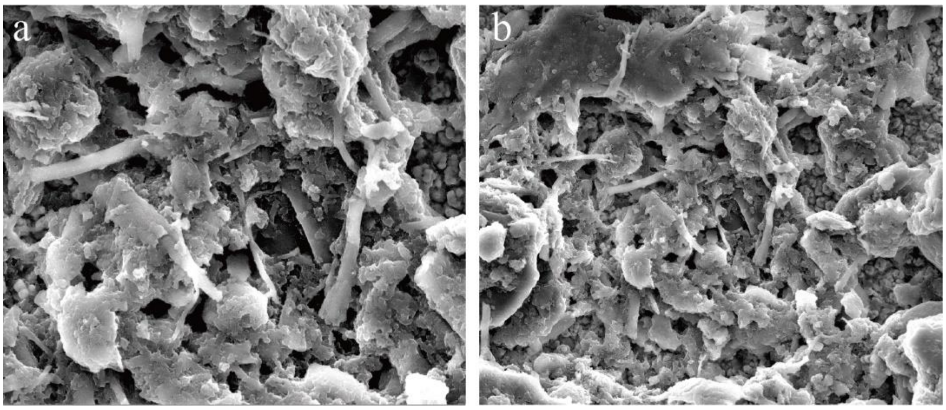

Figure S6. SEM images of FP-C electrodes in LIBs after 20 cycles at current density of 0.5A/g.

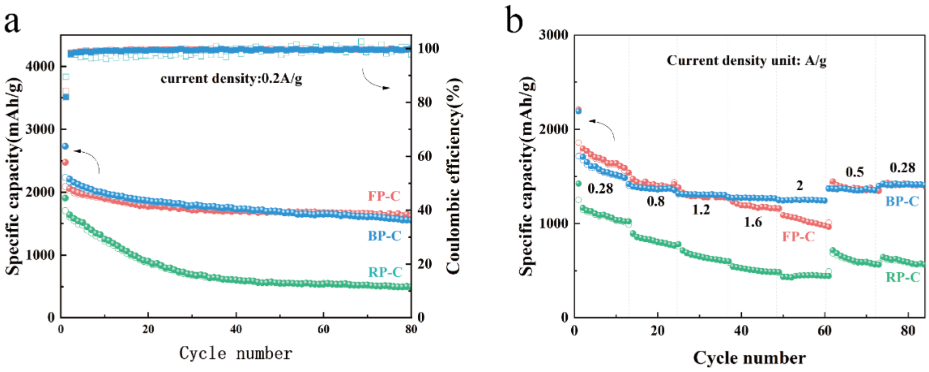

Figure S7. (a)Cycling stability of BP-C anodes for 80 cycles at 0.2A/g.(b) Rate performance of FP-C and RP-C anodes at the varied rate from 0.28A/g to 2A/g.
